# Streamlined instrument-free lysis for the detection of *Candida auris*

**DOI:** 10.1038/s41598-023-47220-7

**Published:** 2023-12-09

**Authors:** Mei Jin, Alexander Y. Trick, Marissa Totten, Pei-wei Lee, Sean X. Zhang, Tza-Huei Wang

**Affiliations:** 1 Department of Biomedical Engineering, Johns Hopkins University, Baltimore, MD USA; 2 Prompt Diagnostics LLC, Baltimore, MD USA; 3 Division of Microbiology, Department of Pathology, Johns Hopkins School of Medicine, Baltimore, MD USA; 4 Department of Mechanical Engineering, Johns Hopkins University, Baltimore, MD USA; 5 Institute of NanoBioTechnology, Johns Hopkins University, Baltimore, MD USA

**Keywords:** Biomedical engineering, Infectious-disease diagnostics, PCR-based techniques

## Abstract

The continued spread of *Candida auris* in healthcare facilities has increased the demand for widely available screening to aid in containment and inform treatment options. Current methods of detection can be unreliable and require bulky and expensive instruments to lyse and identify fungal pathogens. Here, we present a quick, low-cost, instrument-free method for lysis of *C. auris* suitable for streamlined sample processing with polymerase chain reaction (PCR) detection. Chemical, thermal, and bead beating lysis techniques were evaluated for lysis performance and compatibility with nucleic acid extraction and downstream PCR reactions. Using only 10 s of manual shaking with glass beads, this method demonstrated a limit of detection (LOD) of *C. auris* at 500 colony forming units per mL, a 20-fold improvement compared to the LOD without manual shaking, and a 60-fold reduction in time compared to common fungal lysis kits, all while maintaining repeatability and reproducibility across multiple users. This work highlights a simple method for increasing sensitivity and reducing turnaround time of PCR-based *C. auris* detection and exhibits promise for integration into point-of-care platforms towards real-time triage of colonized patients.

## Introduction

Since its emergence in 2009, *Candida auris* has spread to over 40 countries^1,2^. Just within the United States, the CDC reported a 95% annual increase in *C. auris* cases in 2021^3^. Among the growing class of multi-drug resistant organisms, *C. auris* is highly drug resistant, with 90% of isolates resistant to fluconazole, 40% to amphotericin B, and 2% to echinocandins^4^. Echinocandins are currently the recommended first line treatment for *C. auris*. However, with the emergence of echinocandin-resistant *C. auris*^5^, treatment options can be limited, so detection is becoming increasingly crucial for the containment of *C. auris* to prevent further infection in patients or contamination on equipment. Once in hospitals or long-term care facilities, *C. auris* is easily spread because it can survive past fever temperatures (40 °C), remain viable on plastic surfaces for up to 14 days, and spread patient to patient through environmental sources, such as cloth key lanyards or temperature probes^6–11^. Because *C. auris* can colonize people and surfaces for extended periods of time, there is a desperate need for accessible identification methods with rapid turnaround times to increase screening of patient and environmental samples for effective containment.

Current culture-based methods for *C. auris* isolation are slow and insensitive. Despite specialized growth media and conditions, phenotypic methods to identify *C. auris* are insufficient for high accuracy identification^12–14^. Commercial identification platforms relying on biochemical reactions of growing organisms such as API 20C AUX (bioMérieux, Marcy l’Etoile, France), BD Phoenix (BD Diagnostics, Sparks, MD), Vitek-2 (bioMérieux), and MicroScan (Beckman Coulter, Pasadena, CA) have misidentified *C. auris* as various closely related species^15^, which can lead to inappropriate treatment recommendations. An accurate method to identify *C. auris* is matrix-assisted laser desorption/ionization-time of flight mass spectrometry (MALDI-TOF MS), though it requires updated libraries to accurately identify *C. auris*, sample incubation times up to 48 h, and large expensive instruments^14,16,17^. Many community hospitals lack a mycology laboratory, so samples often must be sent to centralized laboratories, which further extends the turnaround time^4^.

Polymerase chain reaction (PCR) detection methods offer fast results for applications where timely identification and containment is necessary. Past studies using PCR based detection methods have demonstrated high sensitivity and specificity for *C. auris*^18,19^. However, prior to PCR detection, cells must be lysed to release genomic DNA for extraction and purification. Fungal lysis methods typically use bead beating protocols that require lengthy procedures with multiple centrifugation steps and specialized vortexing equipment^20^. Some portable cell lysis kits (e.g. Omnilyse) allow operators to mechanically lyse samples without bulky external equipment; however, the cost per sample is expensive, and kits require continuous manual operation for a minute per sample. In this work, the need for easy and fast lysis for detection of *C. auris* was addressed with a single-tube streamlined sample preparation and PCR protocol, requiring only 10 s of manual operation without external equipment for lysis followed by integrated nucleic acid extraction with magnetic beads. The recovery of nucleic acids with chemical, heat, and mechanical lysis methods was compared to that of a commercial kit currently in use in clinical settings. Materials and mechanical lysis conditions were optimized to maximize DNA recovery with minimal PCR inhibition. Lastly, sustained improvement in lysis efficiency and nucleic acid recovery when using manual shaking was demonstrated across multiple users and the limit of detection (LOD) of 500 colony forming units (CFU) per mL was determined.

## Methods

### C. auris culture and quantification

*C. auris* isolate AR0381 (CDC & FDA Antibiotic Resistance Isolate Bank) was stored, incubated, and plated as previously described in Lee et al.^21^. *C. auris* colonies were scraped from the plate and resuspended in phosphate buffered saline (PBS) pH 7.4 1X (Gibco, Paisley, UK) or Liquid Amies medium (RMBIO, Missoula, MT, USA). Using a CO8000 Cell Density Meter (WPA Biowave), the solution was diluted to OD600 value of 0.1, approximately corresponding to 3 × 10^6^ CFU/mL^22–24^, and was further diluted to concentrations 1 × 10^2^–1 × 10^5^ CFU/mL depending on the experiment.

### Primers and PCR reaction

Primer and probe sequences for *C. auris* were adopted from Leach et al. and targeted the ITS2 region^19^. Primer oligos and FAM-labeled Taqman probes were purchased from Integrated DNA Technologies (IDT, Coralville, IA, USA). The PCR buffer consisted of 1 × Lyo PCR Master Mix, dU (Apto-Gen, London, UK), 0.5 uM forward primer, 0.5 uM reverse primer, and 0.1 uM of FAM probe. The PCR buffer containing albumin was prepared using the above concentrations with the addition of 0.8 mg/mL recombinant albumin (NEB, Ipswich, MA, USA). Each reaction was performed on a Bio-Rad CFX96 Touch Real-Time PCR Detection System (Bio-Rad, Hercules, CA) with 1 min 95 °C hotstart, then 40–45 cycles of denaturation (5 s at 95 °C) and annealing (20 s at 60 °C). Positive controls included 1 pg of AR0381 extracted genomic DNA in 10 µL of PCR buffer. No template controls (NTC) included 1 µL of PCR grade water (Quality Biological, Gaithersburg, MD, USA) spiked into 10 µL of PCR buffer.

### Investigation of different lysis methods (Fig. 2)

Samples (1 × 10^5^ CFU/mL of *C. auris* suspended in PBS) processed through the Zymo commercial kit were lysed using the Quick-DNA Fungal/Bacterial Miniprep Kit (Irvine, CA, USA) following the manufacturer’s instructions. Briefly, tubes were loaded with the maximum recommended sample volume (200 µL) and vortexed on the maximum intensity for 10 min with a Vortex Genie 2 (Scientific Industries, Bohemia, NY, USA) using a horizontal microtube holder. Via a series of spin columns, the sample was mixed and eluted with the provided lysis buffer, pre-wash buffer, wash buffer, and elution buffer. The minimum recommended volume (35 µL) of elution buffer was added at the last step to maximize the concentration in the resulting eluent. 1 µL of the eluent was spiked into 10 µL of PCR buffer.

Samples lysed through chemical, heat, or mechanical lysis methods were processed by first mixing 250 µL of the sample (1 × 10^5^ CFU/mL of *C. auris* suspended in PBS), 250 µL of the binding buffer (4 M guanidine thiocyanate (GuSCN) (Sigma Aldrich, St. Louis, MO, USA), 0.055 M Tris–HCl 1 pH 7.5 (Quality Biological, Gaithersburg, MD, USA) 0.025 M pH 8 EDTA (Mediatech, Manassas, VA, USA)), and 10 µL of SeraSil-Mag™ 400 silica coated magnetic beads (Cytiva, USA) in 2 mL polypropylene tubes (DNA Lo-Bind, Eppendorf, Hamburg, Germany). All replicates used the same diluted sample. If processed via mechanical lysis, tubes were pre-filled with 250 µL of zirconia/silica beads (0.5 mm, Cat. No. 11079105z, BioSpec Products), weighed out according to the manufacturers’ listed density. Heat lysis conditions were heated at 70 °C for 10 min before the addition of binding buffer and magnetic beads and allowed to incubate for another 10 min. Mechanical lysis samples were vortexed on the Vortex Genie 2 on maximum intensity for 10 min before the addition of binding buffer and magnetic beads and allowed to sit for an additional 10 min. Chemical lysis samples were allowed to bind at room temperature for 10 min. To decouple effects of the binding buffer from heat or mechanical lysis, the heating or vortexing step was performed before the addition of the binding buffer. Following the binding period, the magnetic beads were pelleted on the side of the tube utilizing a small handheld neodymium magnet (K&J Magnetics, Pipersville, PA) and the tube was inverted to remove remaining contents. The magnetic beads were resuspended in 50 µL wash solution (20% w/v poly(ethylene glycol) (PEG) (Sigma-Aldrich, Germany) 0.05% v/v Tween® 20 (Sigma-Aldrich, St. Louis, MO, USA)). The magnetic beads were pelleted and the wash solution was aspirated. The wash step was repeated for a total of two washes. The magnetic beads were eluted in 10 µL of PCR buffer at 60 °C for 5 min. Three replicates were conducted for each condition.

### Mechanical lysis optimization and evaluation (Fig. 3, 4, 5 and 6)

In subsequent experiments (Fig. 3, 4, 5 and 6), mechanical lysis was conducted by combining the 250 µL lysing beads, 250 µL sample (3 × 10^6^ CFU/mL of *C. auris* suspended in Amies buffer), 250 µL binding buffer, and 10 µL magnetic beads in 1.5 mL polypropylene tubes (DNA Lo-Bind, Eppendorf, Hamburg, Germany) to combine lysis and binding into one step. The Zymo kit was nearing the limit of detection when using 1 × 10^5^ CFU/mL concentration, so subsequent experiment sample concentrations were increased to 3 × 10^6^ CFU/mL. The same washing and elution procedure described above was followed.

### Lysis bead material and PCR buffer optimization (Fig. 3)

For the material optimization experiment, the following beads were tested: glass (Soda Lime Glass, 0.5 mm, Cat. No. 11079105, Biospec Products), yttria-stabilized zirconia (Product Number BA0130, 0.5 mm, MSE Supplies), zirconia (0.7 mm, Cat. No. 11079107zx, BioSpec Products), and zirconia/silica. Conditions with different bead materials were subjected to 1 min of vortexing on maximum intensity. Following lysis and extraction, the magnetic silica beads were subjected to one of two elutions: with and without recombinant albumin. Technical triplicates were conducted for each condition.

### Mechanical lysis time analysis (Fig. 4)

For the vortex time evaluation experiment, all tubes contained glass beads and were vortexed on the Genie Vortexer 2 on maximum intensity for 0, 10, 30, or 60 s. Mechanical lysis via manual shaking was performed by shaking the tube longitudinally so that the beads would collide with the bottom and cap of the tube for 0, 10, 30, or 60 s. Technical triplicates were conducted for each condition.

### User shaking variability analysis (Fig. 5)

For user variability analysis, the 10 s shaking step was performed by 4 different operators, and all other steps in the assay were performed by a single operator to isolate the effect of different shaking techniques. Technical triplicates were conducted for each operator.

### LOD determination (Fig. 6)

For the LOD analysis experiment, samples of *C. auris* were suspended in Amies transport media at concentrations of 3 × 10^6^, 1 × 10^5^, 1 × 10^4^, 1 × 10^3^, 5 × 10^2^, 2 × 10^2^, and 1 × 10^2^ CFU/mL. Technical triplicates were performed for concentrations of 1 × 10^4^—3 × 10^6^ CFU/mL. Technical replicates of 6 were performed for concentrations of 1 × 10^2^—1 × 10^3^ CFU/mL. NTC conditions used 250 µL of Amies as the “sample”. The “shaking” tubes were shaken for 10 s and contained sample, glass beads, binding buffer, and magnetic silica beads. The “no shaking” tubes only contained sample, binding buffer, and magnetic silica beads.

### Data analysis

**Figure 1:**
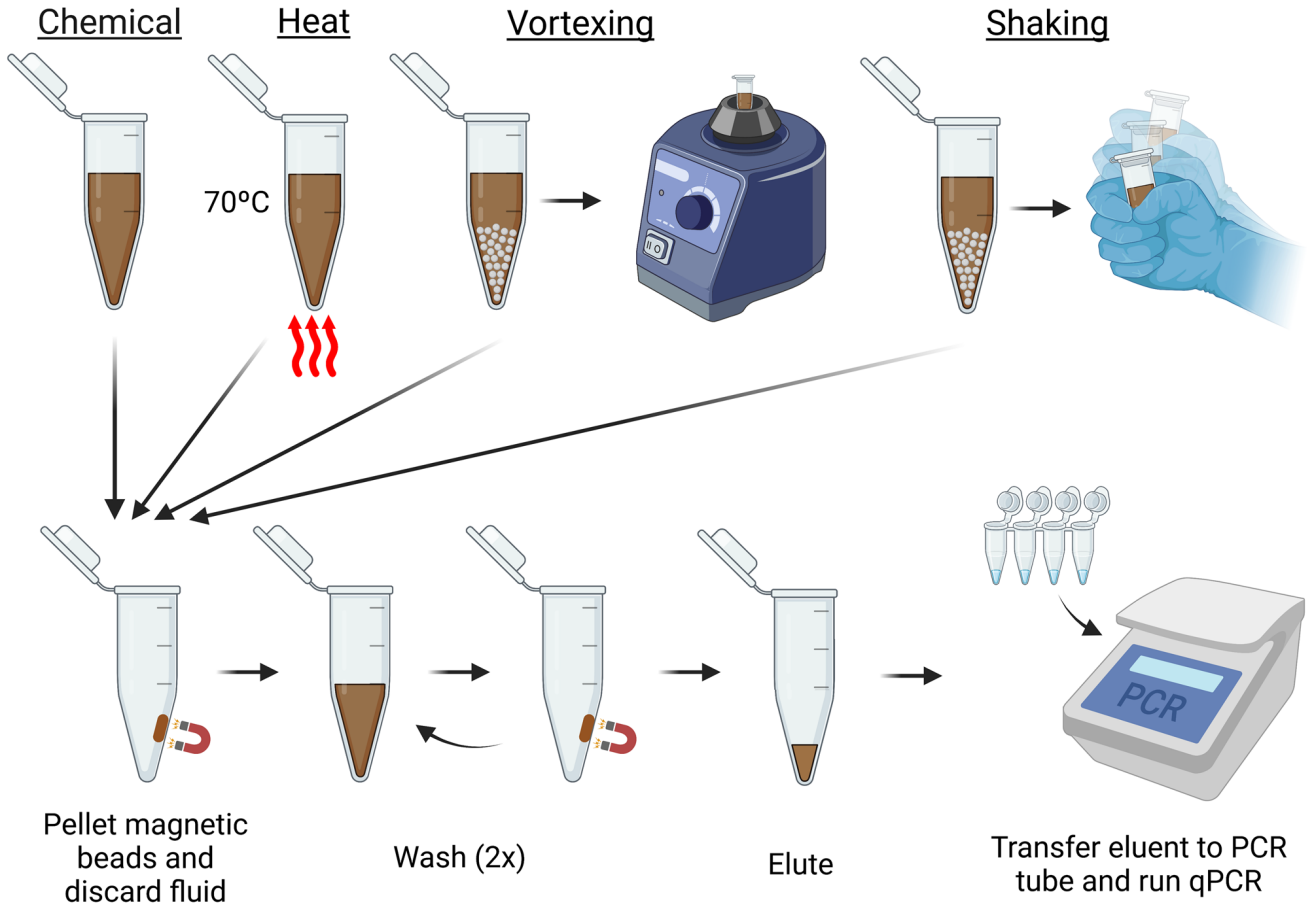
Workflow of lysis and nucleic acid extraction techniques. Following one of the lysis methods (top row), all samples were subjected to the same wash and PCR buffer elution protocol in the original tube used for lysis (bottom row).

**Figure 2:**
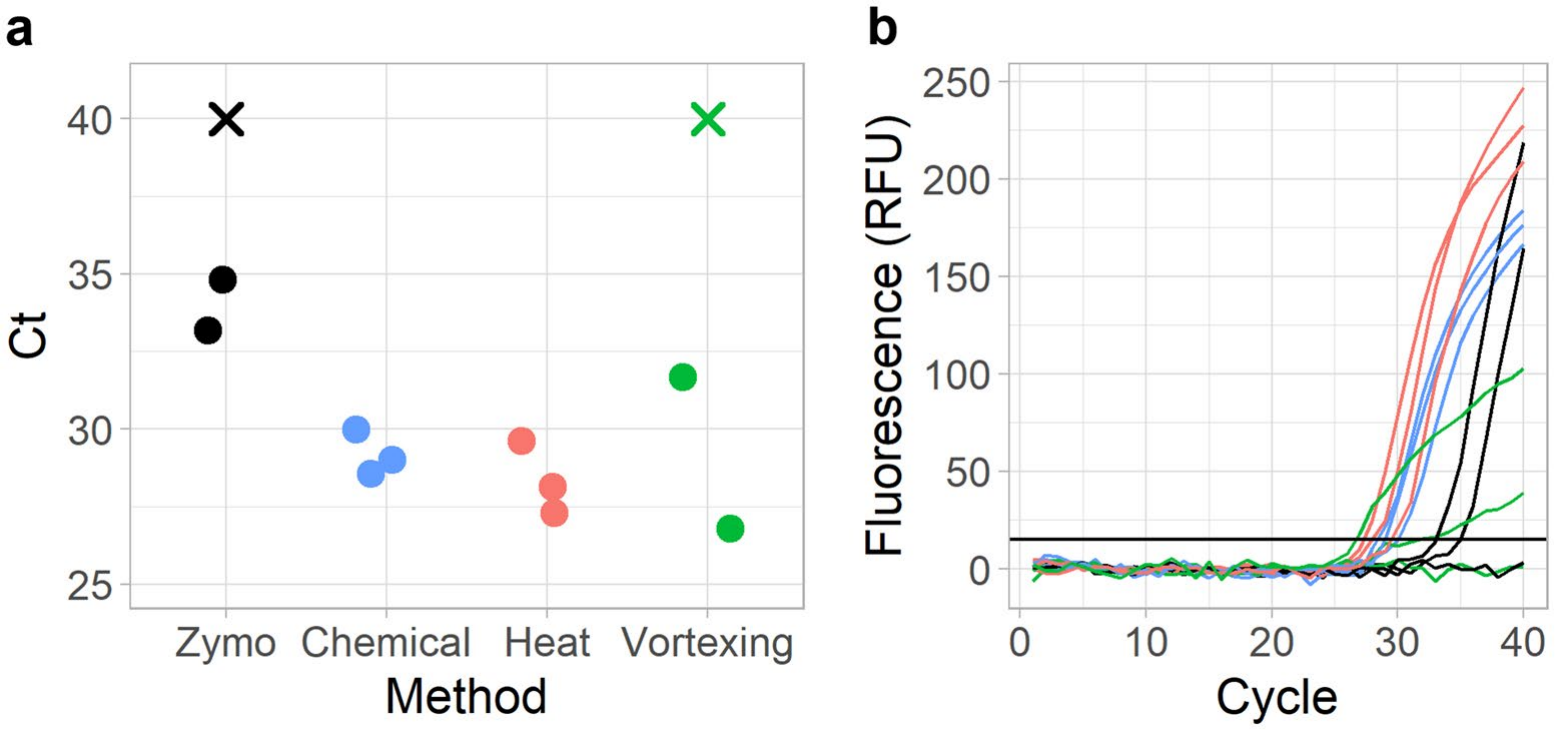
Comparison of Zymo, chemical, heat, and vortexing lysis and purification protocols. (**a**) *C*_*t*_ values based on the lysis method. X’s indicate no amplification. n = 3. (**b**) Real-time PCR fluorescence. Horizontal line indicates the *C*_*t*_ threshold.

**Figure 3:**
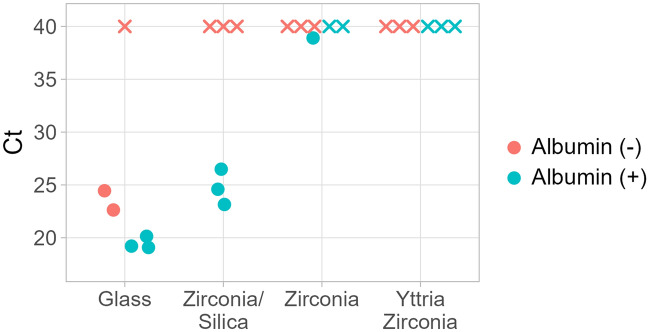
*C*_*t*_ values of bead beating material with and without the addition of recombinant albumin to the PCR mix. X’s indicate no amplification. n = 3.

**Figure 4:**
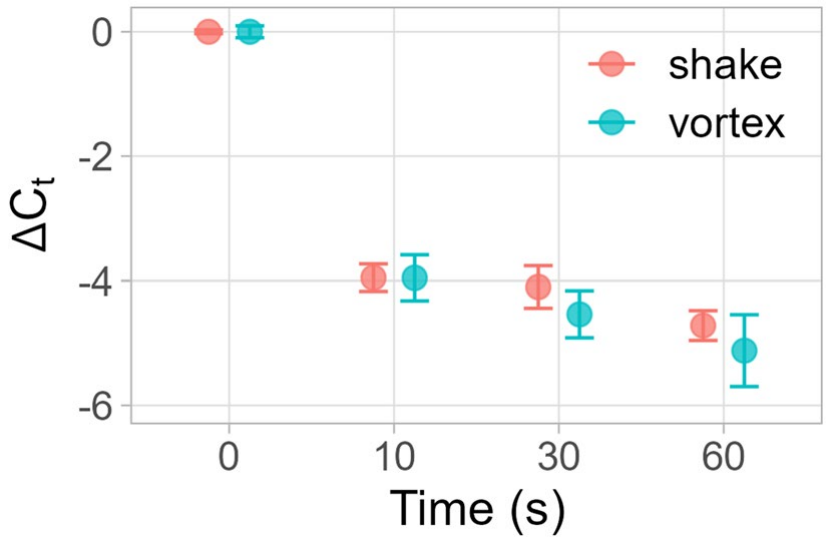
Change in cycle threshold means (Δ*C*_*t*_) of samples with varying duration of vortexing or manual shaking compared to no mechanical lysis. Error bars indicate standard error of difference of means. n = 3.

**Figure 5:**
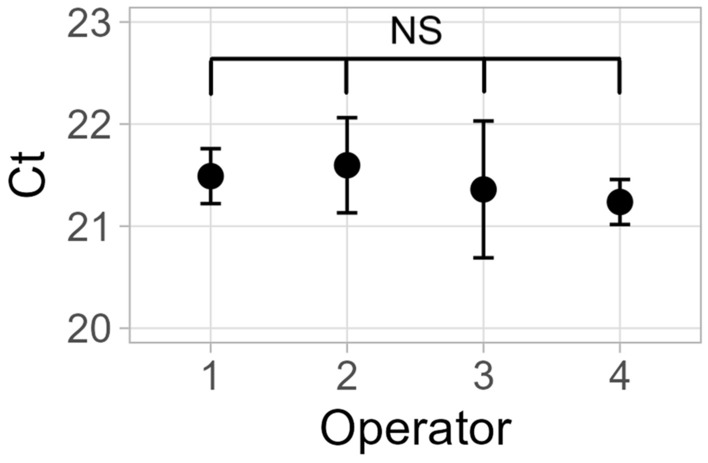
User variability in hand shaking for mechanical lysis of C. auris. Error bars indicate standard deviation. One-way ANOVA test indicated no significant difference (F = 0.373). n = 3 tubes per operator.

**Figure 6:**
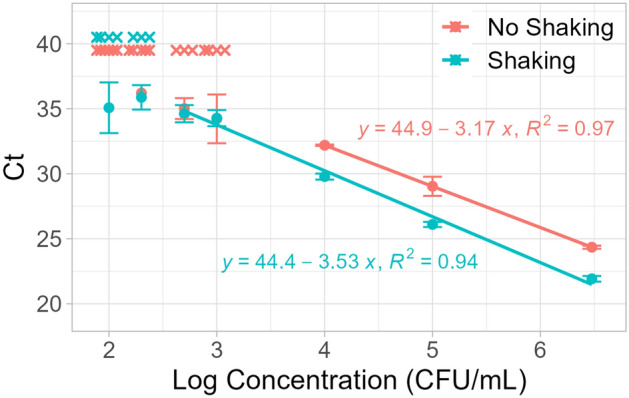
LOD analysis. X indicates no amplification. Circles indicate mean *C*_*t*_. Error bars indicate standard error. n = 3 for concentrations 1 × 10^4^—3 × 10^6^ CFU/mL. n = 6 for concentrations 1 × 10^2^—1 × 10^3^ CFU/mL.

Cycle threshold (*C*_*t*_) values were determined through the Bio-Rad CFX Manager software (version 3.1) using a threshold fluorescence value of 15 relative fluorescence units (RFU) across all trials. Statistical values such as mean, standard deviation, and standard error were computed on R software (version 4.1.2). One-way ANOVA statistical testing for user variability and linearity calculations were performed on R. Figures were created with BioRender.com (Fig. 1) and generated using R (Figs. 2, 3, 4, 5 and 6).

## Results and discussion

We first investigated various lysis methods to explore which method would lead to the highest DNA recovery. We compared guanidine thiocyanate (GuSCN) based chemical lysis, heat lysis, and vortexer based bead beating to a commercial kit currently used in the Johns Hopkins Hospital mycology laboratory (Zymo Quick-DNA Fungal/Bacterial) (Fig. 1). Heat lysis was investigated because previous work has demonstrated heat lysis temperature optimization of *C. auris* ^25^, which can be conducted with minimal instrumentation (i.e. simple heat block).

The commercial kit yielded *C*_*t*_ values that were approximately 6 cycles later than any of the in-house methods (Fig. 2a,b). This low recovery for the commercial kit can be attributed to the small 1 µL fraction out of 35 µL of total eluent spiked into the PCR reaction. Conversely, the custom in-house methods provided higher DNA recovery per reaction by directly eluting into the PCR buffer. For the silica-bead based extraction methods, the addition of a heating step before extraction led to a minor, yet statistically insignificant improvement in *C*_*t*_ (Δ*C*_*t*_ = 0.81, *p*-value = 0.3542) when compared to chemical lysis only (Fig. 2a). One replicate of bead beating mechanical lysis yielded the earliest *C*_*t*_ of all trials, which was promising as an indicator that mechanical lysis by vortexing could yield the highest recovery of DNA. However, mechanical lysis also resulted in flatter slopes and lower endpoints, suggesting bead beating introduced inhibitors into the PCR reaction (Fig. 2b).

Due to the promise of early amplification from bead beating and past work demonstrating the efficacy of bead beating for nucleic acid recovery^26,27^, different bead materials and PCR buffer recipes were tested to improve the amplification profile and the consistency of bead beating results. Inhibitory effects have been shown to be dependent on the bead beating material used and to be mitigated with the addition of bovine serum albumin (BSA)^20^. We tested four commonly used bead beating materials for fungal cells: glass, zirconia/silica, zirconia, and yttria-stabilized zirconia, and we tested two different PCR buffers, with and without recombinant albumin. The addition of recombinant albumin to the PCR buffer resulted in earlier *C*_*t*_ values or increased the number of detected samples (Fig. 3). When comparing the glass bead trials with and without albumin, the amplification curves with the albumin had a much steeper slope, indicating that the albumin sequestered inhibitors that were released during vortexing (Fig. S1). Of all the materials, glass had the lowest *C*_*t*_ values and steepest amplification slopes (Fig. 3, Fig. S1). Glass also showed the least improvement in amplification with the addition of albumin, indicating that vortexing glass beads released the least amount of inhibitors. Therefore, we continued to evaluate bead beating variables utilizing glass lysis beads and PCR buffer containing albumin in further experiments.

The effect of vortexing time on nucleic acid recovery was evaluated from 0 to 60 s. With just 10 s of vortexing, *C*_*t*_ values shifted 4 cycles earlier compared to 0 s of vortexing, roughly corresponding to a 16-fold improvement in DNA recovery (Fig. 4). Increasing time from 10 to 60 s led to a 1.17 *C*_*t*_ improvement at the cost of additional processing time, flatter amplification profiles, and reduced endpoint signal. (Fig. 4, Fig. S2).

We wanted to investigate an instrument-free procedure of mechanical lysis by manually shaking the tubes. Manual shaking lysis would lower costs, save time, and negate the need for additional equipment in comparison to other commercial methods such as Omnilyse and Zymo Fungal/Bacterial Miniprep kit (Table 1). Omnilyse, a single-use portable device that lyses cells as the user pushes and pulls a syringe to pass the sample solution through a miniature motor, has a high cost per unit, making application in low-resource settings unfeasible. Zymo, the aforementioned widely-used commercial DNA extraction kit, requires a vortexer and centrifuge to operate, which is not suitable to the point-of-care. Therefore, simple manual shaking offers an attractive alternative for testing in low-resource settings.

**Table 1:** Comparison of material costs, lysis operation time, and instrumentation requirements of different mechanical lysis methods.

Feature	Manual Shaking	Omnilyse	Zymo
Cost per sample	$0.25	$11.25	$2.00
Time	10 s	< 1 min	10 min
Instrument-free	✔	✖	✖

Like the vortexing results, manual shaking improved *C*_*t*_ values by 4 cycles with just 10 s of shaking, with marginal improvement at higher durations (Fig. 4, Fig. S2). Shaking lysis was maintained at 10 s in subsequent testing to limit operator fatigue.

Because manual shaking speed is subject to user variability during operation, the effect different operators had on the consistency of the assay was evaluated. Four different operators each shook three separate tubes for 10 s each. Statistical testing (one-way ANOVA) determined that there were no significant differences in *C*_*t*_ values between operators, despite a large variation between operators in number of shakes completed within that 10 s timeframe (Fig. 5, Fig. S3). Therefore, manual shaking was determined to be a robust and reproducible process and was not operator dependent.

After establishing that improvement in DNA recovery from manual shaking was reproducible, the LOD of the assay with and without manual shaking was evaluated. LOD is defined to be the lowest concentration at which all replicates amplify. We tested concentrations of 3 × 10^6^, 1 × 10^5^, 1 × 10^4^, 1 × 10^3^, 5 × 10^2^, 2 × 10^2^, and 1 × 10^2^ CFU/mL. The LOD with no shaking was determined to be 1 × 10^4^ CFU/mL while the LOD with manual shaking was determined to be 5 × 10^2^ CFU/mL (Fig. 6), which indicates roughly a 20 times higher sensitivity. This improved LOD agrees with earlier testing that showed a 4-cycle improvement, or approximately 16 times higher DNA recovery.

## Conclusion

This work demonstrated an instrument-free mechanical lysis method for releasing *C. auris* genomic DNA. Compared to the fungal lysis and DNA extraction efficiency of a commercial kit, chemical lysis, and heat lysis, mechanical lysis was the most promising. Glass beads were found to be the bead beating material with the least PCR inhibition, likely due to its superior resistance to fragmentation and release of PCR inhibiting particles. The addition of albumin was demonstrated to be key to consistent amplification and maintaining a steep amplification profile. Using glass beads and PCR buffer containing albumin, the minimum vortexing time needed to achieve significant sensitivity was found to be 10 s. For possible application in low-resource settings, bead beating without the need for a vortexer was investigated and only 10 s of manual shaking was necessary to see a 16-fold improvement in DNA recovery. This increased sensitivity from manual shaking was reproducible across multiple users. Lastly, the LOD of manual shaking was determined to be 500 CFU/mL whereas the LOD of chemical lysis without shaking was 10,000 CFU/mL, corresponding to a 20 times improvement in LOD. Previous works have demonstrated an LOD at the same order of magnitude while using freeze-heating and homogenizers ^19^ (200 CFU/mL) and Omnilyse^21^ (300 CFU/mL) lysis methods, which indicates that the lysis performance of manual shaking in conjunction with the our silica bead DNA extraction results in sensitivity on par with commercially available methods.

While we have demonstrated efficient lysis and nucleic acid extraction without the need for specialized vortexing equipment and centrifuges, one limitation of this work is the requirement of a PCR thermocycler for the final detection. To truly enable point-of-care testing, integration of our method into automated portable platforms will be assessed. These platforms execute both magnetic extraction of DNA and PCR detection in less than 30 min and have been demonstrated in low-resource settings^28–31^. Even though all experiments performed in this study were performed manually, all components of the assay, including the magnetic silica beads, GuSCN binding buffer, and the PEG wash, have previously been integrated into automated magnetofluidic cartridges^28^. Another limitation of this study is tested samples were spiked culture. Future testing of this platform will be performed using clinical samples from patients or environmental swabs and will be compared to current clinical tests, such as culture followed by MALDI-TOF MS. The integration of the shaking lysis technique to previously developed point-of-care platforms will help expand *C. auris* testing capabilities beyond well-resourced laboratories toward resource-limited local laboratories and even toward bedside testing.

Furthermore, this platform could be extended to antifungal susceptibility testing (AFST). Whereas current laboratory standard culture-based methods require three to seven days to generate results^32–34^, which is too long to inform treatment options, PCR based genotypic assays that evaluate AFST through known resistance genes offer rapid and accurate results^35^ and are easily integrated with our lysis and DNA extraction method.

With *C. auris* becoming a widespread public health concern in healthcare facilities, it is important to expand testing and increase accessibility. This manual shaking bead beating method combined with single-tube DNA extraction offers a simple, affordable, and quick alternative method to increase sensitivity of *C. auris* detection, offering a step toward more accessible diagnostic tools to healthcare facilities to inform treatment decisions and containment measures.

### Supplementary Information

Supplementary Information.

## Data Availability

The datasets generated during and/or analyzed during the current study are available from the corresponding author on reasonable request.
