# Supplementary material for: Streamlined instrument-free lysis for the detection of Candida auris
Source: Sci Rep. 2023 Dec 9;13:21848. doi: 10.1038/s41598-023-47220-7 (PMC10710429; doi:10.1038/s41598-023-47220-7)
Supplement: Supplementary file 1 — Supplementary Information. [file 41598_2023_47220_MOESM1_ESM.pdf]

Streamlined instrument-free lysis for the detection of Candida auris

Mei Jin, Alexander Y. Trick, Marissa Totten, Pei-wei Lee, Sean X. Zhang, Tza-Huei Wang

Supplemental Figures

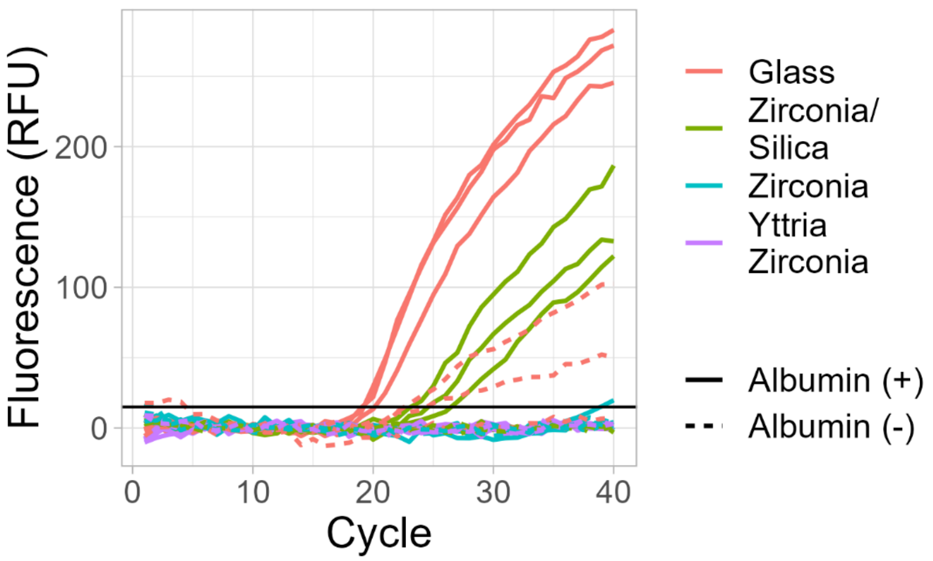

Figure S1. Amplification curves for comparison of different bead beating materials and the presence of albumin in the PCR buffer. The black horizontal line indicates the C_t threshold value.

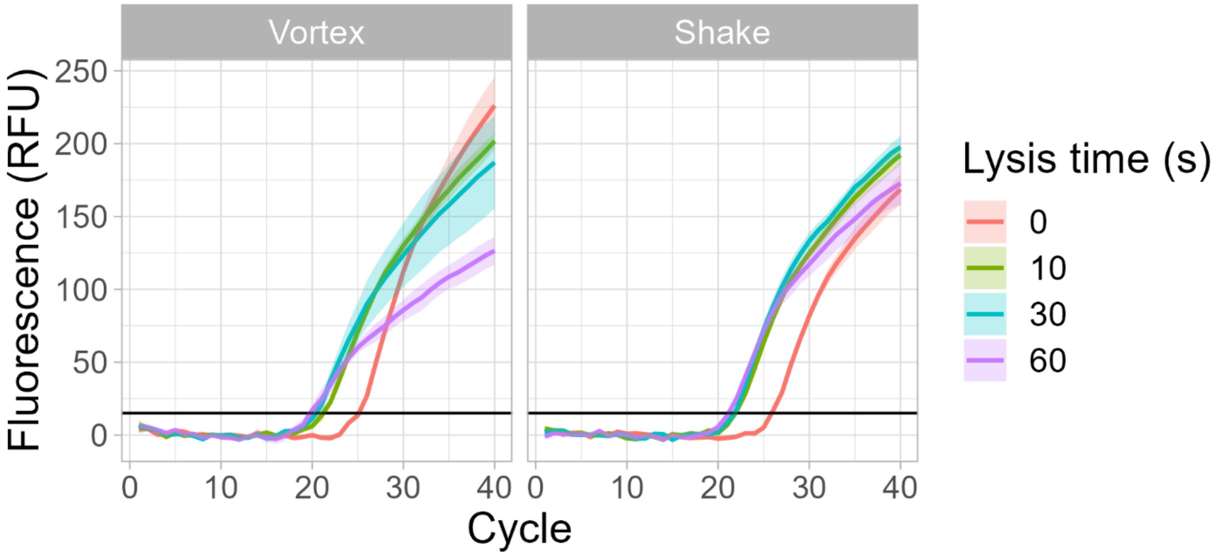

Figure S2. Amplification curves for time optimization for (**a**) vortexing and (**b**) shaking. Lines indicate averaged fluorescence profiles and shaded area indicate standard error. The black horizontal line indicates the C_t threshold value.

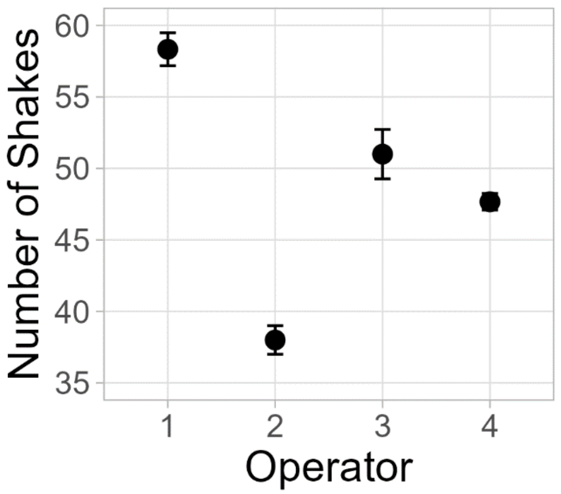

Figure S3. Average number of shakes for each operator with 10 seconds of shaking. Error bars indicate standard deviation.
